# Experimental and Theoretical Investigation of the Thermophysical Properties of Cobalt Oxide (Co_3_O_4_) in Distilled Water (DW), Ethylene Glycol (EG), and DW–EG Mixture Nanofluids

**DOI:** 10.3390/nano12162779

**Published:** 2022-08-13

**Authors:** Monther Alsboul, Mohd Sabri Mohd Ghazali, Mohamed R. Gomaa, Aliashim Albani

**Affiliations:** 1 Physics Department, College of Science, Al Hussein Bin Talal University, Maan 71111, Jordan; 2 Faculty of Science and Marine Environment, Universiti Malaysia Terengganu, Kuala Terengganu 21030, Malaysia; 3 Mechanical Engineering Department, Faculty of Engineering, Al Hussein Bin Talal University, Maan 71111, Jordan; 4 Mechanical Engineering Department, Benha Faculty of Engineering, Benha University, Benha 13512, Egypt; 5 Renewable Energy & Power Research Interest Group (REPRIG), Eastern Corridor Renewable Energy (ECRE), Faculty of Ocean Engineering Technology and Informatics, Universiti Malaysia Terengganu, Kuala Terengganu 21030, Malaysia

**Keywords:** cobalt oxide nanoparticles, thermal conductivity, viscosity, density, DW–EG mixture nanofluids

## Abstract

Solid particles scattered in a base fluid for a standard no larger than 100 nm, constituting a nanofluid, can be used to improve thermophysical characteristics compared to the base fluid. In this study, theoretical and experimental investigations were carried out to estimate the density, viscosity, and effective thermal conductivity of Co_3_O_4_ in distilled water (DW), ethylene glycol (EG), and DW–EG mixture nanofluids. Co_3_O_4_ nanoparticles with diameters of 50 nm were dispersed in different base fluids (i.e., EG, DW, 60EG:40DW, 40EG:60DW, 20EG:80DW) with varying concentrations of 0.025–0.4 vol.%. Thermal conductivity was estimated by the hot-wire technique, and viscosity was determined using a viscometer apparatus. According to the measurements, the viscosity of Co_3_O_4_ nanofluids decreased with increasing temperature, and increased with increasing volume fraction. The results revealed that the thermal conductivity of Co_3_O_4_ nanofluids increased with increasing temperature and volume concentrations. Moreover, the measurements found that the maximum thermal conductivity of 10.8% and the maximum viscosity of 10.3% prevailed at 60 °C in the volume fraction of 0.4%. The obtained viscosity and thermal conductivity results of the present experiments on Co_3_O_4_ nanofluids were compared with previous results. The results showed good agreement with theoretically proposed models to predict nanofluids’ viscosity and thermal conductivity. Thus, the thermal conductivity results of Co_3_O_4_ nanofluids are promising with respect to the use of nanofluids in solar thermal applications.

## 1. Introduction

Nanofluids are colloidal systems in which nanoscale particles are suspended in a liquid. Investigating nanometer-sized particles and their influence on the thermophysical properties and transport properties of suspensions is an active research area. It was found that atypical properties such as thermal conductivity or viscosity changes are dependent on the size of the system. Several reasons for this exist, including particle size, particle type, and the state of particle aggregation in suspension Not only their chemical composition determines their properties, as several studies in the literature have described this behavior for nanofluids that are derived from metallic nanoparticles, metal oxide nanoparticles, ceramic nanoparticles, or carbon nanotubes [1,2,3,4,5,6,7,8,9]. In a previous study [10], the authors compiled a collection of articles reporting on the thermal conductivity of different nanofluids, showing that it depends on factors such as volume fraction, size, and shape of the nanoparticles, their morphology, additives, pH, temperature, type of base liquid, and nanoparticle material. This poses a major problem in terms of sample characterization and reproducibility of experimental data. The literature review reveals significant discrepancies between different thermal conductivity data sets reported for this system, where general consensus shows that the distinctions are based on a combination of factors, such as a variety of preparation processes, sample stability, particle size distribution, non-uniformity of particle shape, clustering, sedimentation, and pH [10,11,12,13]. In addition to their transport properties, nanofluids also show unusual behavior in terms of their viscoelastic properties [1,14,15,16,17,18,19,20,21], and this represents a challenge not only due to the difficulties associated with their experimental determination, but also because of the very limited knowledge about the underlying physicochemical phenomena that might justify the observed trends. Co_2_O_3_, Co_3_O_4_, and CoO nanoparticles are commonly used in catalysis [22,23], drug delivery [24], wastewater treatment [25], hyperthermia [26], and data storage media [27]. In the field of supported magnetic nanoparticles, cobalt oxide (Co_3_O_4_) nanoparticles are of particular interest, due to their applications in lithium batteries [28], in various catalytic reactions [29], and in the aerobic oxidation of alcohols [30], among other uses. In another study [31], it was pointed out that the literature does not contain sufficient experimental data on nanofluids’ specific heat, density, and viscosity; the authors emphasized the importance of developing reliable databases. The magnetic and electrical properties of dry Co_3_O_4_ nanoparticles have been investigated [32,33,34,35], whereas Co_3_O_4_ nanoparticles dispersed in liquids have, to date, only been considered in a limited number of studies [36,37]. For instance, Vickers et al. [36] found that suspensions of Co_3_O_4_ nanocubes in oligomeric polyethylene glycol (PEG) behave similarly to Newtonian liquids at low particle volume fractions, but exhibit complex rheological behavior at higher particle volume fractions, including shear thinning and shear thickening. Using Co_3_O_4_ nanoparticles dispersed in paraffin and oleic acid as the capping agent, Hosseini et al. [37] examined the effects of nanoparticle concentration on the rheological properties resulting from dispersion. They also found that not all samples followed Newtonian dynamics.

The use of nanofluids to cool electronic components is also being investigated [38,39]. There are different magnetic fields within electronic components, across which nanofluids must flow. Several studies have shown that magnetic fields affect heat transfer rates in nanofluids containing magnetic particles [40]. We aimed to investigate the effect of magnetic fields on the heat transfer rate of a magnetic Co_3_O_4_ nanofluid in an EG–DW mixture. Therefore, magnetic Co_3_O_4_ nanoparticles were used for the preparation of nanofluids in this study. In order to assess nanofluid heat transport, we needed to determine the thermophysical properties of these fluids.

Based on the literature review, several metal- and metal-oxide-based nanofluids lack thermophysical properties. The following materials are of particular importance: vanadium (V), chromium (Cr), nickel (Ni), molybdenum (Mo), tungsten (W), zinc (Zn), and niobium (Nb) [41], for the following reasons:

1. Visible light is absorbed by transition metals and their oxides with band gaps smaller than 2.5 eV (such as Mn and Co).
2. UV light can be transmitted through transition metals and their oxides with band gaps greater than 3.5 eV (e.g., Hf, Zr, Ce, Nd, Er, Dy).

Therefore, this study examines nanofluids containing Co_3_O_4_ nanoparticles dispersed in nanofluids of distilled water (DW), ethylene glycol (EG), and DW–EG mixtures at concentrations up to 0.4% by volume. These properties are very useful in designing nanofluid-based heat-exchange devices. This paper attempts to provide data on thermal conductivity, viscosity, and density to further investigate heat transfer characteristics. Thermal conductivity was measured using the hot-wire technique. The experimental values were compared with the theoretical values for the various properties studied in terms of volume fraction and temperature.

## 2. Methodology

### 2.1. Preparation of Nanomaterial Samples

The Co_3_O_4_ nanoparticles were bought from Sigma-Aldrich, USA. The Co_3_O_4_ nanoparticles were used as received from the manufacturer, without any further purification. The EG was supplied by Tedia (99%). The suspension was prepared by mixing powdered Co_3_O_4_ nanoparticles and a DW–EG mixture. The physical properties of the nanoparticles (cobalt oxide) and base fluids (DW and EG) are shown in Table 1 [42].

The preparation procedure of Co_3_O_4_ nanofluids was conducted as follows: The first step was weighing the amount of the Co_3_O_4_ nanopowder required for the solid volume fraction in the base fluid, using a digital electronic balance (Adam Model AAA 250L). The Co_3_O_4_ nanoparticles needed for the experimental samples of the base fluids were estimated using the following expression.

(1) $$\emptyset =\frac{\frac{w_{np}}{\rho_{np}}}{\frac{w_{bf}}{\rho_{bf}}+\frac{w_{np}}{\rho_{np}}}\times 100\%$$

where ∅ is the volume concentration (%); *w_np_* and *w_bf_* are the weight of the nanoparticles and nanofluids (g), respectively, while $\rho_{np}$ and $\rho_{bf}$ are the density of the nanoparticles and nanofluids (g/cm^3^), respectively.

The base fluid density of EG (1) + DW (2) can be described by the following equation [43]:

(2) $$\rho =w_{1}y_{1}+w_{2}y_{2}+\left(y_{1}-y_{2}\right)w_{1}w_{2}\left(A_{4}+A_{5}w_{1}+A_{6}t\right)$$

where *t = T/K* − 273.15, *w*_1_ is the mass fraction of glycol, *w*_2_ *=* 1 − *w*_1_, and *y*_1_ and *y*_2_ are the ρ-values for pure EG and DW, respectively. In the case of ρ, *y*_1_ is given as follows:

(3) $$y_{1}=A_{1}+A_{2}t+A_{3}t^{2}$$

The coefficients *Ai* of Equations (2) and (3) are shown in Table 2.

The second step was inserting the Co_3_O_4_ nanoparticles into a weighed bade fluid. For about three hours, the experimental samples were subjected to a magnetic stirrer (Wisd Model MSH-20A) to mix the Co_3_O_4_ nanoparticles and the base fluid.

**Figure 1:**
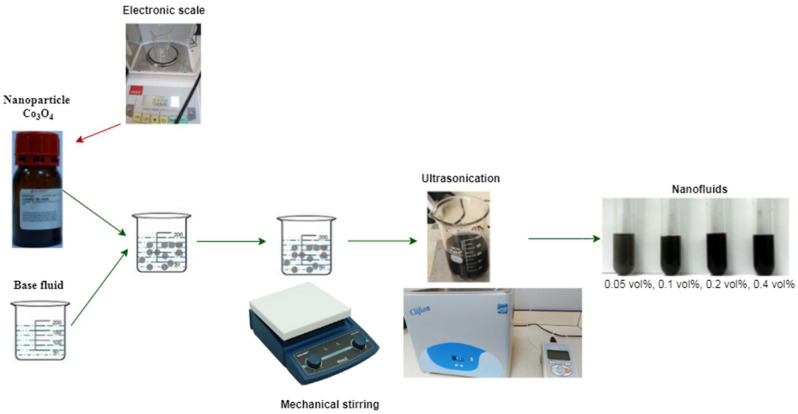
Schematic diagram of the Co_3_O_4_ nanofluid preparation and measurement of its thermophysical properties.

The third step was sonicating the Co_3_O_4_ nanofluid suspensions, which were inserted in the ultrasonic cleaner set, carried out for 40 min using the ultrasonic processor (Wisd WUC-A06H model, power density = 172 watts, frequency = 40 kHz). In their study, Divya et al. [44] indicated that the optimal time of ultrasonication after 40 min clustering of nanoparticles occurred for water-based nanofluids. The two-step technique of preparing and measuring the thermophysical properties of Co_3_O_4_ nanoparticles in distilled water (DW), ethylene glycol (EG), and DW–EG mixture nanofluids is shown in Figure 1. This technique was used to bring down the aggregation of nanoparticles’ sedimentation, prohibit the sedimentation, and obtain a stable dispersion and suspension of nanoparticles.

The mass of Co_3_O_4_ nanoparticles needed to prepare Co_3_O_4_/DW, Co_3_O_4_/EG, and Co_3_O_4_/DW–EG mixture nanofluids of several solid volume fraction concentrations for 50 mL of base fluid is summarized in Table 3. The water-based 0.025%, 0.05%, 0.1%, 0.2%, and 0.4% volume concentrations of Co_3_O_4_ nanofluids were prepared by scattering 0.07640, 0.15286, 0.30580, 0.61220, and 1.11700 g of Co_3_O_4_ nanoparticles, respectively, in 50 mL of water as a base fluid.

### 2.2. Characterization of Co_3_O_4_ Nanoparticles

**Figure 2:**
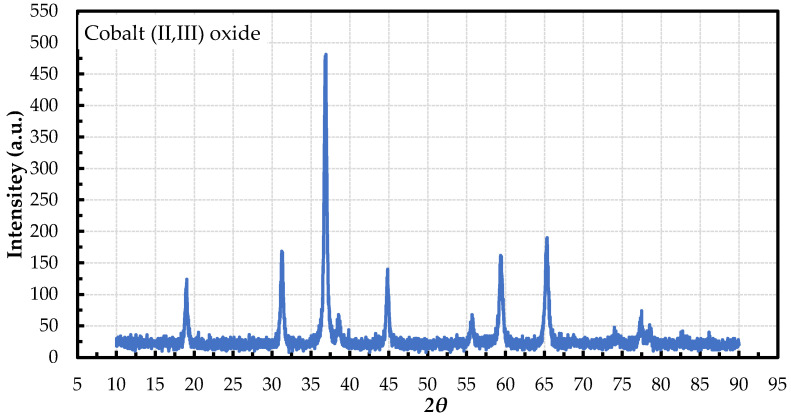
*XRD* patterns of cobalt (*II/III*) oxide nanoparticles.

The *XRD* patterns of synthesized Co_3_O_4_ nanoparticles are shown in Figure 2. The *XRD* pattern of Co_3_O_4_ nanoparticles shows Co_3_O_4_ peaks. The 2*θ* positions of the Co_3_O_4_ sample are 19.04°, 31.24°, 36.8°, 44.84°, 55.5°, 59.38°, and 65.28°, which can be indexed as the 111, 220, 311, 400, 422, 333, and 440 planes, respectively, for Co_3_O_4_ nanoparticles. No clear reflection peaks from other impurities were observed from the spectrum.

### 2.3. Uncertainty Analysis

Instrumentation, data acquisition, and data analysis are among the sources of uncertainty in experimental works [45]. By comparing the experimental results with published data, we first evaluated the accuracy of the instruments used to measure density, viscosity, and thermal conductivity. Moreover, in the present study, density, viscosity, and thermal conductivity were measured at least three times at each point. Experimental uncertainty was calculated according to Moffat’s theory [46,47]. According to Moffat’s theory, *D* is the sum of the results of different measured variables, *Xi*, where *D* = *f* (*X*_1_, *X*_2_, ..., *Xi*). The uncertainty of each variable can be estimated through Equation (4) based on this theory:

(4) $$\frac{U_{D}}{D}=\sqrt{{\left(\frac{\partial X_{1}}{X_{1}}\right)}^{2}+{\left(\frac{\partial X_{2}}{X_{2}}\right)}^{2}+\ldots +{\left(\frac{\partial X_{n}}{X_{n}}\right)}^{2}}$$

Table 4 displays the uncertainty results calculated based on the mean ± standard error for the determination of density, viscosity, thermal conductivity, and convective heat transfer coefficient.

## 3. Theoretical and Experimental Density, Thermal Conductivity, and Viscosity of Nanofluids

### 3.1. Mathematical Model

The effective density of the nanofluid can be calculated analytically with the volume fraction using the mixing theory as follows [48]:

(5) $$\rho_{nf}=\left(1-\emptyset_{p}\right)\rho_{bf}+\emptyset_{p}\rho_{p}$$

where ∅ is the volume concentration (%), $\rho_{nf}$ is the density of the nanofluid (g/cm^3^), $\rho_{p}$ is the density of the nanopowder (g/cm^3^), and $\rho_{bf}$ is the density of the base fluid (g/cm^3^).

Maxwell [49] suggested different classical models. From the literature, including the works of Crosser, Wasp, and Bruggeman, it is possible to estimate the efficient thermal conductivity of liquid–solid suspensions (*k_nf_*). Moreover, Maxwell [49] suggested an equation for estimating the suspension’s *k_nf_*, which is valid for spherical particles in volume fractions of less than 1.0 vol%, and is expressed as follows:

(6) $$k_{nf}=\left[\frac{k_{p}+2k_{bf}+2\left(k_{p-}k_{bf}\right)\emptyset_{p}}{k_{p}+2k_{bf}-\left(k_{p}-k_{bf}\right)\emptyset_{p}}\right]k_{bf}$$

where *k_nf_* is the thermal conductivity of the nanofluid, $\emptyset_{p}$ is the particle volume concentration of the nanoparticles, and *k_bf_* is the thermal conductivity of the base fluid.

Furthermore, the Maxwell–Eucken model [50] proposes an equation to predict the nanofluid’s *k_nf_*, as follows:

(7) $$k_{nf}=k_{bf}\left\{\frac{\left[\left(1+2\emptyset_{p}(1-(k_{bf}/k_{p}\right)))/(2\left(k_{bf}/k_{p}\right)+1)\right]}{\left[(1-\emptyset_{p}\left(1-\left(k_{bf}/k_{p}\right))\right)/(\left(k_{bf}/k_{p})+1\right)\right]}\right\}$$

The Yu and Choi model [51] for the *k_nf_* of suspensions is given as follows:

(8) $$k_{nf}=k_{bf}\left[\frac{k_{p}+2k_{bf}+2\emptyset_{p}\left(k_{p}-k_{bf}\right){\left(1+\beta \right)}^{3}}{k_{p}+2k_{bf}-\emptyset_{p}\left(k_{p}-k_{bf}\right){\left(1+\beta \right)}^{3}}\right]$$

A few experiments on the viscosity of the nanofluids and associated correlations were conducted to predict the nanofluids’ viscosity (*μ_nf_*) with respect to particle volume concentration and base fluid density. Below are a few of the models of *μ_nf_* produced by numerous investigators.

Among the equations used to calculate the *μ_nf_* of a nanofluid is the Einstein model [52]. When the volume concentration of spherical nanoparticles is less than 5%, Einstein’s method expresses it using the following model:

(9) $$\mu_{nf}=\left(1+2.5\emptyset_{p}\right)\mu_{bf}$$

where *μ_nf_* is the viscosity of the nanofluid, $\emptyset_{p}$ is the particle volume concentration of the nanoparticles, and *μ_bf_* is the viscosity of the base fluid. On the other hand, the Brinkman model [53] presents a correlation model for calculating the *μ_nf_* of nanofluids, as shown in Equation (10):

(10) $$\mu_{nf}=\frac{1}{{\left(1-\emptyset \right)}^{2.5}}\mu_{bf}$$

The *μ_nf_* of the nanofluids is determined from the de Batchelor model [54], which is expressed as follows:

(11) $$\mu_{nf}=\left(1+2.5\emptyset +6.2\emptyset^{2}\right)\mu_{bf}$$

### 3.2. Density, Thermal Conductivity, and Viscosity Measurement of Co_3_O_4_/DW, EG, and DW–EG Mixture Nanofluids

Several techniques can be used to estimate the density (*ρ*) of nanofluids, such as the gravimetric technique and the Archimedes method. In the present study, the density of the Co_3_O_4_ nanofluids was investigated five several solid volume fraction concentrations of 0.025, 0.05, 0.1, 0.2, and 0.4%. The volume concentrations under examination were determined using a gravimetric technique (pycnometer), and the experimental results of the density obtained were compared with the results obtained using the density correlation equation (Equation (12)) suggested by Pak and Cho [48] for nanofluids.

The density of Co_3_O_4_/DW, EG, and DW–EG mixture nanofluids was measured at room temperature by weighing a sample of each fluid in a standard 25 mL volumetric flask on an electronic balance with high precision (±0.0001 g). The procedure was repeated three times, and the collected data were averaged. The density was calculated using the following equation:

(12) $$\rho_{nf}=\left[\frac{m_{t}-m_{fl}}{V_{nf}}\right]$$

where $m_{t}$ and $m_{fl}$ are the total mass of the flask with the nanofluid and the mass of the empty flask, respectively, and $V_{nf}$ is the volume of the nanofluid taken in the flask. Volumetric flask calibration was carried out in the experimental condition with distilled water. The accuracy of the instrument was ±5%. We placed the nanofluid sample in the small container and inserted the probe into the center of the container. The instrument had a specified accuracy of ±5%. A temperature range of 20–60 °C with a step size of 5 °C was achieved by immersing the container in the surrounding fluid kept inside the refrigerated/heating circulator, which maintained the surrounding fluid’s temperature within 0.1 °C. The precise results were obtained by continuously holding the probe in the nanofluid sample for 20 min after reaching the desired equilibrium temperature. For each sample, five measurements were taken to ensure repeatability and accuracy.

The base fluid (i.e., distilled water) characteristics, such as density, viscosity, and thermal conductivity, were determined as shown in Equations (13)–(15), respectively, considering the base temperature for regression equations [11].

(13) $$\rho_{w}=1000\times \left[1-\frac{{\left(T_{w}-4.0\right)}^{2}}{119,000+\left(1365\times T_{w}\right)-\left(4\times {\left(T_{w}\right)}^{2}\right)}\right]$$

where $\rho_{w}$ is the density of the distilled water (g/cm^3^), and *T_w_* is the temperature of the distilled water (°C).

(14) $$\begin{array}{c} \mu_{w}=0.00169-4.25263\times 10^{-5}\times \left(T_{w}\right)+4.9255\times 10^{-7}\times {\left(T_{w}\right)}^{2}\\ -2.0993504\times 10^{-9}\times {\left(T_{w}\right)}^{3} \end{array}$$

where *μ_w_* is the distilled water’s viscosity (mPa.s), and *T_w_* is the temperature of the distilled water (°C).

(15) $$\begin{array}{c} k_{w}=0.56112+0.00193\times \left(T_{w}\right)-2.60152749\times 10^{-6}\times {\left(T_{w}\right)}^{2}\\ -6.08803\times 10^{-8}\times {\left(T_{w}\right)}^{3} \end{array}$$

where $k_{w}$ is the thermal conductivity of the distilled water (W/mK), and *T_w_* is the distilled water’s temperature (°C).

Several methods can be used to evaluate the effective thermal conductivity of nanofluids, such as the transient hot-wire, parallel steady-state plate, and cylindrical cell methods. In the present study, the transient hot-wire technique (KD2 Pro) was extended by using a KD2 Pro instrument, due to its high speed and precision in measurement. The effective thermal conductivity of Co_3_O_4_/DW, Co_3_O_4_/EG, and Co_3_O_4_/DW–EG mixture nanofluids was studied at various solid volume fractions and temperatures. The KD2 Pro was calibrated utilizing glycerin. Table 5 shows the conductivity meter properties.

In this study, we used the KD2 Pro apparatus to measure the thermal conductivity of the nanofluids. The KD2 premeasured the heat transfer properties of the low-temperature fluids without causing any convection, via a principle of measurement based on the transient hot-wire method, as described by several authors [13,55].

The KD2 Pro analyses the different thermophysical properties—including thermal conductivity, resistivity, diffusivity, and specific heat—with the use of a resilient heat transfer method, namely, the line heat-source method. The KD2 Pro apparatus generally consists of three different measuring inputs with different needles and temperature sensors. The needles serve as both temperature sources and sensor equipment. The thermal conductivity of the nanofluid was considered under temperatures ranging from 25 °C to 50 °C. The different concentrations of the nanofluids (0.05% to 0.4%) were subjected individually to temperatures of 20 °C, 25 °C, 30 °C, 35 °C, 40 °C, 45 °C, 50 °C, 55 °C, and 60 °C. The sensor was integrating into the interior heating element and thermo-resistor. It was connected to a microprocessor for controlling the controller module, containing a battery, a 16-bit microcontroller/AD converter, and power control circuitry. Each measurement cycle consisted of 90 s. During the first 30 s, the instrument equilibrated, which was then followed by heating and cooling the sensor needles for 30 s each. At the end of the reading, the controller computed the thermal conductivity using the temperature change (DT)–time data. The estimated uncertainty of thermal conductivity measurement was lower than 3%. Previous studies have discussed the advantages of this technique when applied to nanofluids. The calibration of the sensor needle was carried out first by measuring the thermal conductivity of distilled water, glycerin, and ethylene glycol. The measured values for distilled water, glycerin, and ethylene glycol were 0.611, 0.292, and 0.263 (W/mK), respectively, which are consistent with the literature values of 0.613, 0.285, and 0.252 (W/mK), respectively, within ±5% accuracy [56,57].

In the present analysis, the transient hot-wire system was implemented due to its high speed, and measurement precision was used to calculate the effective thermal conductivity of the Co_3_O_4_/DW, Co_3_O_4_/EG, and Co_3_O_4_/DW–EG mixture nanofluids using a KD2 Pro instrument with a KS-1 sensor. This sensor is ideal for measuring the effective thermal conductivity of various nanofluid types with different base fluids. The effective thermal conductivity of the Co_3_O_4_/DW, Co_3_O_4_/EG, and Co_3_O_4_/DW–EG mixture nanofluids was measured at various solid volume fractions and temperatures.

The effective thermal conductivity of the samples of Co_3_O_4_ nanofluids was calculated three times, and the final values were taken as the average.

Several methods are used to evaluate nanofluids’ kinetic viscosity, viscosity, and static viscosity, including capillary, vibrational, and rotational methods. In the present study, the rotational process was extended by using an A&D Vibro Viscometer (SV-10, Tokyo, Toshima Cit) instrument with a range measurement from 0.3 and 10 Pa.s. Empirical data were taken with an interval of 5 °C.

A few experiments on the viscosity of the nanofluids and associated correlations were established to predict nanofluid viscosity (*μ_nf_*) with respect to particle volume concentration and base fluid density. The experiments aimed to investigate the impacts of the Co_3_O_4_/DW, Co_3_O_4_/EG, and Co_3_O_4_/DW–EG mixture nanofluids’ temperature and volume on their *μ_nf_*. The A&D Vibro Viscometer (SV-10, Japan) was utilized for measuring the viscosity of the Co_3_O_4_/DW, EG, and DW–EG mixture nanofluids. The measurements were carried out in the temperature range from 20 °C to 60 °C. The calibration of the viscometer was carried out with calibration liquids.

Co_3_O_4_ nanofluids at five different volume concentrations of 0.025, 0.05, 0.1, 0.2, and 0.4% were prepared for measuring the temperature-dependent viscosity of all of the nanofluids and concentrations considered in this work.

## 4. Results and Discussion

### 4.1. Experimental Density, Thermal Conductivity, and Viscosity of Co_3_O_4_/EG, DW, and EG–DW Mixture Nanofluids

#### 4.1.1. The Experimental Density of Co_3_O_4_/EG, DW, and EG–DW Mixture Nanofluids

**Figure 3:**
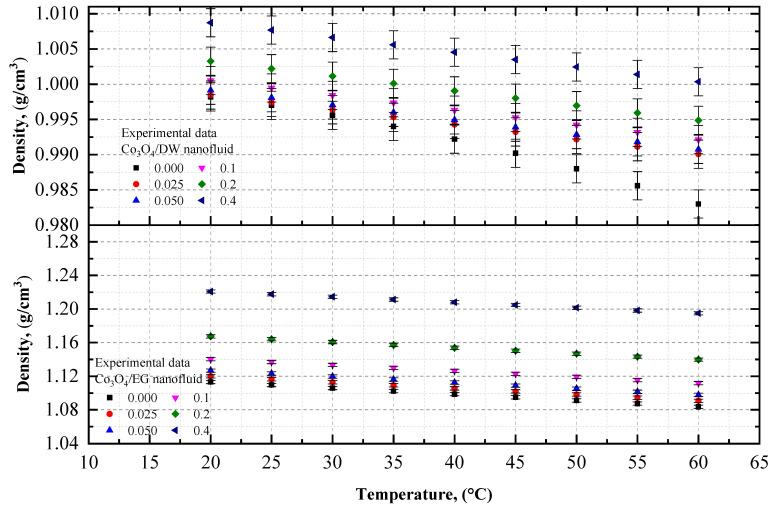
Experimental density values for various volume concentrations of Co_3_O_4_/EG and Co_3_O_4_/DW nanofluids with respect to temperature.

**Figure 4:**
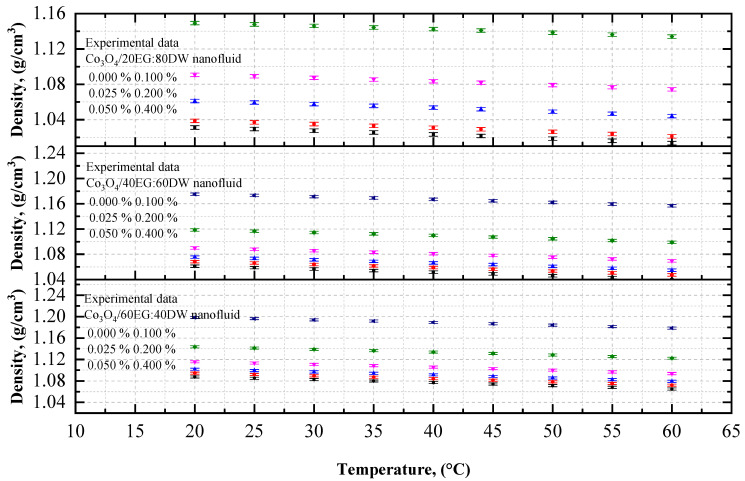
Experimental values of density for various volume concentrations of Co_3_O_4_/EG–DW mixture nanofluids with respect to temperature.

Experimental data on the density of the Co_3_O_4_ nanoparticles with EG and DW, or with 20EG:80DW, 40EG:60DW, and 60EG:40DW, as base fluids are shown in Figure 3 and Figure 4, respectively, at different temperatures, with a 5 °C increment, and various volume concentrations. The density of the Co_3_O_4_/EG, Co_3_O_4_/DW, and Co_3_O_4_/EG–DW mixture nanofluids increased linearly with increasing volume concentrations from 0.025% to 0.4%, and decreased with increasing temperatures from 20 °C to 60 °C. The DW-based nanofluid had the lowest value of density (0.99 g/cm^3^) at *T* = 60 °C and volume concentration = 0.025%, and the EG-based nanofluid had the highest value of density (1.22 g/cm^3^) at *T* = 20 °C and volume concentration = 0.4%.

As shown in Figure 3, the density of the DW-based Co_3_O_4_ nanofluid was enhanced by 0.004% and 1.02%, whereas the density of the EG-based Co_3_O_4_ nanofluid was improved by 0.61% and 9.68%, at 0.025% and 0.4% volume concentrations at *T* = 20 °C, respectively in comparison with the density of the base fluids (DW and EG).

As shown in Figure 4, the density of the 20EG:80DW-based Co_3_O_4_ nanofluid was enhanced by 0.73% and 11.48% at 0.025% and 0.4% volume concentrations at *T* = 60 °C, respectively, compared to the density of the base fluid (20EG:80DW). Similarly, the density of the 40EG:60DW-based Co_3_O_4_ nanofluid was enhanced by 0.68% and 10.78% at 0.025% and 0.4% volume concentrations at *T* = 60 °C, respectively, compared to the density of the base fluid (40EG:60DW). In contrast, the density of the 60EG:40DW-based Co_3_O_4_ nanofluid was enhanced by 0.64% and 10.21% at 0.025% and 0.4% volume concentrations at *T* = 60 °C, respectively, in comparison to the density of the base fluid (60EG:40DW). Under different base fluids, we observed that the density enhancement was lesser at low volume concentrations of 0.025% and temperature of 60 °C, while it was higher at high volume concentrations of 0.4% and temperatures of 20 °C.

The experimental density values of the Co_3_O_4_ nanoparticles with EG, DW, 20EG:80DW, 40EG:60DW, and 60EG:40DW as base fluids, at 5 °C intervals from 20 °C to 60 °C, are listed in Table 6.

#### 4.1.2. Experimental Viscosity of Co_3_O_4_/DW, Co_3_O_4_/EG, and Co_3_O_4_/EG–DW Mixture Nanofluids

**Figure 5:**
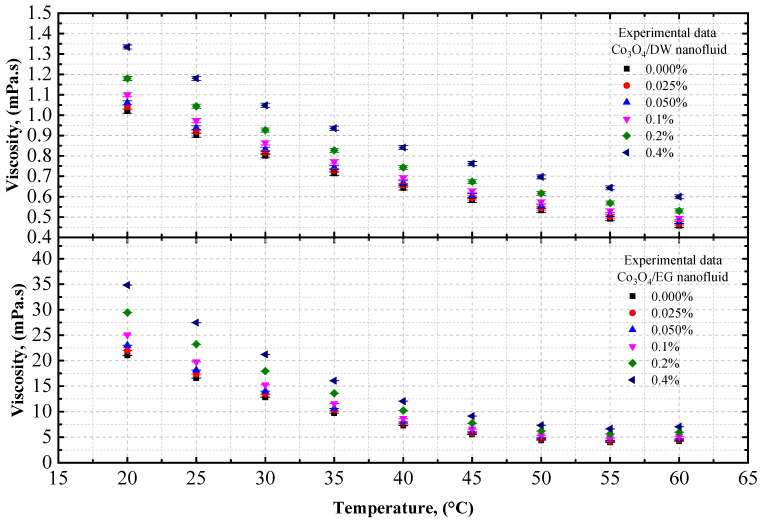
Experimental viscosity values for various volume concentrations of Co_3_O_4_/DW and Co_3_O_4_/EG nanofluids with respect to temperature.

**Figure 6:**
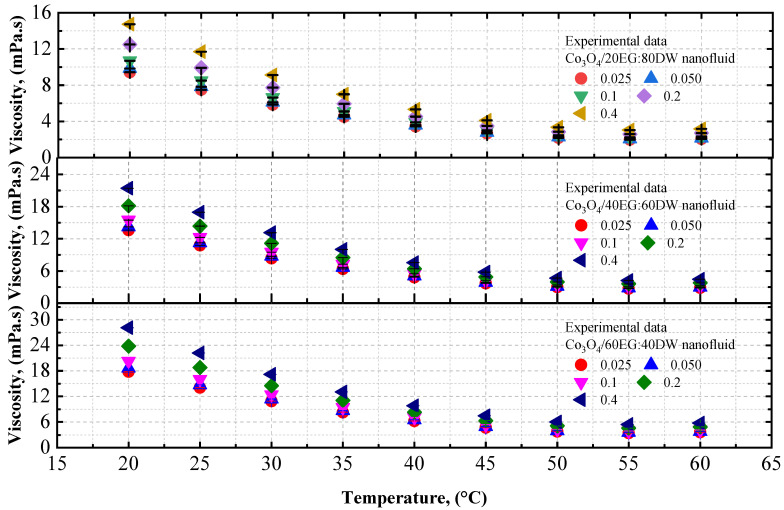
Experimental viscosity values for various volume concentrations of Co_3_O_4_/EG–DW mixture nanofluids with respect to temperature.

The viscosity data of the Co_3_O_4_/EG and Co_3_O_4_/DW nanofluids, and of the Co_3_O_4_/20EG:80DW, 40EG:60DW, and 60EG:40DW nanofluids, are shown in Figure 5 and Figure 6, respectively, at different temperatures (temperature increment of 5 °C per step) and volume concentrations. The viscosity of the Co_3_O_4_/EG, Co_3_O_4_/DW, and Co_3_O_4_/DW–EG mixtures increased with increasing volume concentrations and decreased with increasing temperatures from 20 °C to 60 °C. The DW-based nanofluid had the lowest viscosity (0.467 mPa.s) at *T* = 60 °C and volume concentration = 0.025%, while the EG-based nanofluid had the highest viscosity (22 mPa.s) at *T* = 20 °C and volume concentration = 0.4%.

As shown in Figure 5, the viscosity of the DW-based Co_3_O_4_ nanofluid was enhanced by 0.06% and 0.96%, whereas the viscosity of the EG-based Co_3_O_4_ nanofluid was improved by 0.01% and 0.92%, at 0.025% and 0.4% volume concentrations at *T* = 20 °C, respectively, in comparison with the base fluids (DW and EG).

As shown in Figure 6, the viscosity of the 20EG:80DW-based Co_3_O_4_ nanofluid was enhanced by 1.16% and 2.07% at 0.025% volume concentrations and 0.4% volume concentrations at *T* = 20 °C, respectively, compared to base fluid (20EG:80DW). Similarly, the viscosity of 40EG:60DW -based Co_3_O_4_ nanofluid is enhanced by 0.70% and 1.61% at 0.025% and 0.4% volume concentrations at *T* = 20 °C, respectively, compared to the base fluid (40EG:60DW). On the other hand, the viscosity of the 60EG:40DW-based Co_3_O_4_ nanofluid was enhanced by 7.45% and 6.54% at 0.025% and 0.4% volume concentrations at *T* = 20 °C, respectively, in comparison to the base fluid (60EG:40DW).

The experimental viscosity values of the Co_3_O_4_ nanoparticles with EG, DW, 20EG:80DW, 40EG:60DW, and 60EG:40DW as base fluids, at 5 °C intervals from 20 °C to 60 °C, are listed in Table 7.

#### 4.1.3. Experimental Thermal Conductivity of Co_3_O_4_/EG, Co_3_O_4_/DW, and Co_3_O_4_/EG–DW Mixture Nanofluids

**Figure 7:**
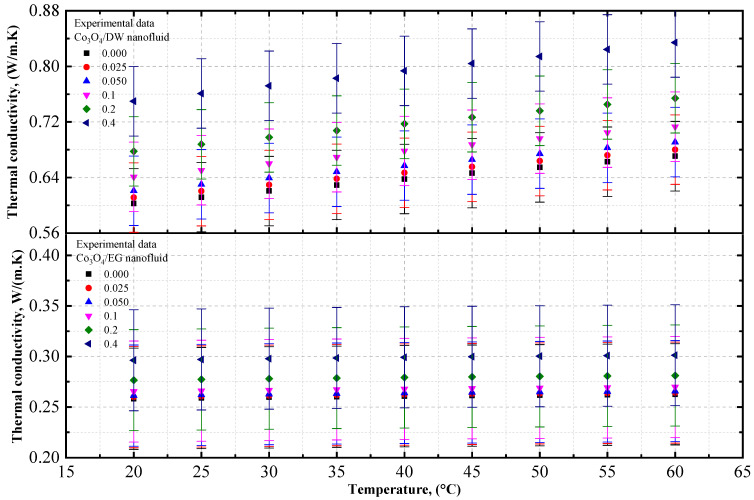
Experimental thermal conductivity values for various volume concentrations of Co_3_O_4_/DW and Co_3_O_4_/EG nanofluids with respect to temperature.

**Figure 8:**
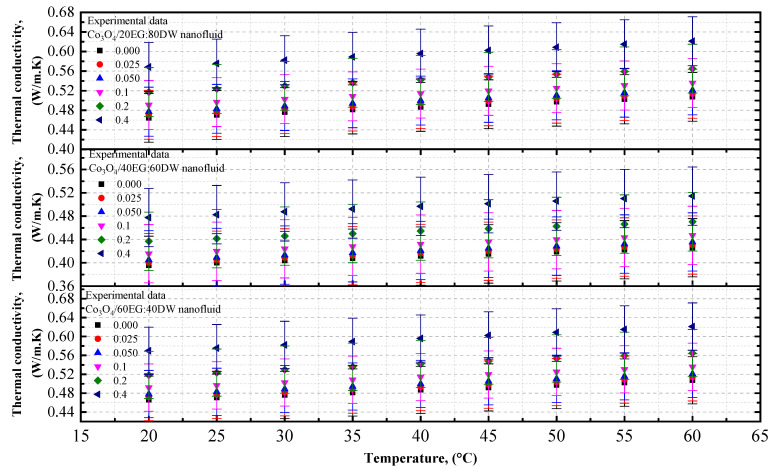
Experimental thermal conductivity values for various volume concentrations of Co_3_O_4_/EG–DW mixture nanofluids with respect to temperature.

The thermal conductivity data of the Co_3_O_4_/EG and Co_3_O_4_/DW nanofluids, and of the Co_3_O_4_/20EG:80DW, 40EG:60DW, and 60EG:40DW nanofluids, are shown in Figure 7 and Figure 8, respectively, at different temperatures (in increments of 5 °C/step) and volume concentrations. It can be observed that the thermal conductivity of the Co_3_O_4_/EG, DW, and EG–DW mixture nanofluids increased with increasing volume concentrations and increasing temperatures from 20 °C to 60 °C. The EG-based nanofluid had the lowest thermal conductivity value of 0.259 W/m·K at *T* = 20 °C and a volume concentration of 0.025%. The DW-based nanofluid had the highest thermal conductivity value of 0.834 W/m·K at *T* = 60 °C and a volume concentration of 0.4%. As shown in Figure 7, the thermal conductivity of the DW-based Co_3_O_4_ nanofluids was enhanced by 1.04% and 24. 4%, whereas the thermal conductivity of the EG-based Co_3_O_4_ nanofluid was enhanced by 0.61% and 14.07%, at 0.025% and 0.4% volume concentrations at *T* = 20 °C, respectively, in comparison with the base fluids (DW and EG).

Figure 8 shows that the thermal conductivity of 20EG:80DW-based Co_3_O_4_ nanofluid was enhanced by 0.123% and 22.2% at 0.025% and 0.4% volume concentrations at *T* = 20 °C, respectively, compared to the base fluid (20EG:80DW). Similarly, the thermal conductivity of the 40EG:60DW-based Co_3_O_4_ nanofluid was enhanced by 0.1327% and 22.3% at 0.025% and 0.4% volume concentrations at *T* = 20 °C, respectively, compared to the base fluid (40EG:60DW). On the other hand, the thermal conductivity of the 60EG:40DW-based Co_3_O_4_ nanofluid was enhanced by 0.017% and 20.69% at 0.025% and 0.4% volume concentrations at *T* = 20 °C, respectively, compared to the base fluid (60EG:40DW).

The experimental thermal conductivity values of the Co_3_O_4_ nanoparticles with EG, DW, 20EG:80DW, 40EG:60DW, and 60EG:40DW as base fluids, at 5 °C intervals from 20 °C to 60 °C, are listed in Table 8.

### 4.2. Experimental Density, Thermal Conductivity, and Viscosity Comparison of Co_3_O_4_/DW and Co_3_O_4_/EG Nanofluids

**Figure 9:**
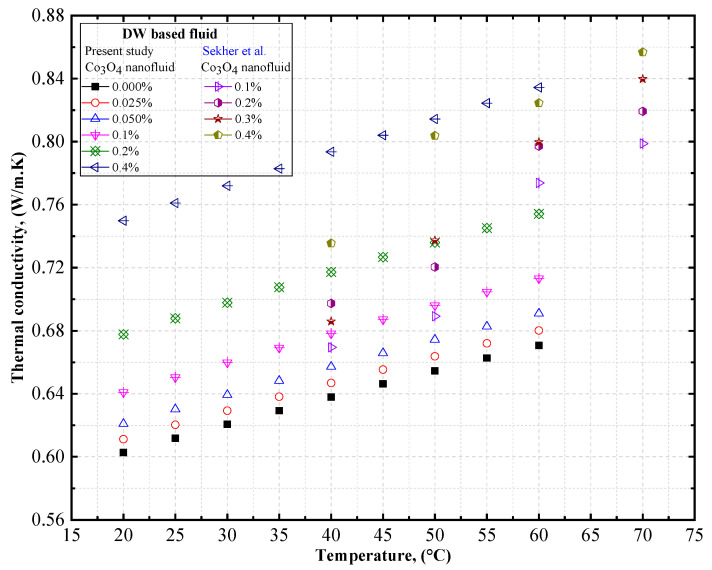
Comparison of our experimental data on the thermal conductivity of DW-based Co_3_O_4_ nanofluids. Reprinted with permission from Ref. [58], 2022, Elsevier.

To verify the accuracy of our measurements, the thermal conductivity of DW-based Co_3_O_4_ nanofluids was measured at different temperatures, and was compared with the data obtained by Sekhar et al. [58]. Figure 9 compares the experimental thermal conductivity of the DW-based Co_3_O_4_ nanofluids derived from the present study with the data obtained by Sekhar et al. [58] for Co_3_O_4_ nanofluids. Similar trends of increasing thermal conductivity with a rise in the concentration of the solid volume fraction and increasing thermal conductivity with an increase in temperature were observed by Sekhar et al. [58] with DW-based Co_3_O_4_ nanofluids (Table 9).

**Figure 10:**
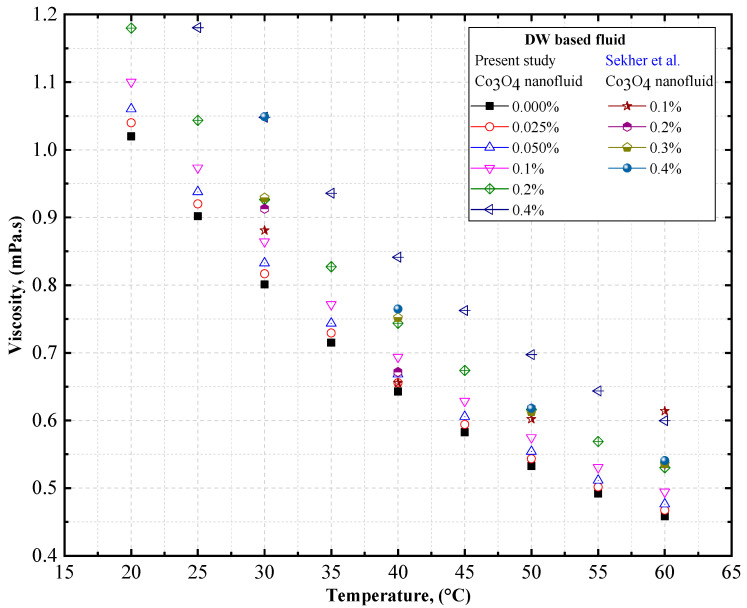
Comparison of experimental data on the viscosity of DW-based Co_3_O_4_ nanofluids. Reprinted with permission from Ref. [58], 2022, Elsevier.

To verify the accuracy of our measurements, the viscosity of DW-based Co_3_O_4_ nanofluids was measured at different temperatures, and was compared with the data obtained by Sekhar et al. [58]. Figure 10 compares the experimental thermal conductivity of the DW-based Co_3_O_4_ nanofluids derived from the present study with the data obtained by Sekhar et al. [58] for Co_3_O_4_ nanofluids. Similar trends of increasing viscosity with an increase in the concentration of the solid volume fraction and decreasing viscosity with an increase in temperature were observed by Sekhar et al. [58] with DW-based Co_3_O_4_ nanofluids (Table 10).

**Figure 11:**
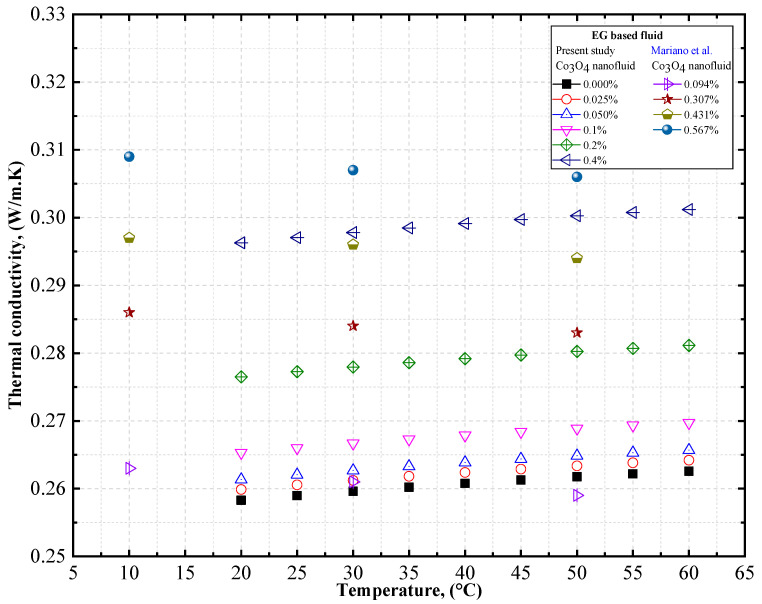
Comparison of experimental data on the thermal conductivity of EG-based Co_3_O_4_ nanofluids. Reprinted with permission from Ref. [59], 2022, Elsevier.

To verify the accuracy of our measurements, the thermal conductivity of DW-based Co_3_O_4_ nanofluids was measured at different temperatures, and was compared with the data obtained by Mariano et al. [59]. Figure 11 compares the experimental thermal conductivity of EG-based Co_3_O_4_ nanofluids derived from the present study with the data obtained by Mariano et al. [59] for Co_3_O_4_ nanofluids. A similar trend of increase in thermal conductivity with a rise in the concentration of the solid volume fraction was observed by Mariano et al. [59] in Co_3_O_4_/EG nanofluids. However, a decrease in thermal conductivity with an increase in temperature was observed by Mariano et al. [59] for EG-based Co_3_O_4_ nanofluids (Table 11).

**Figure 12:**
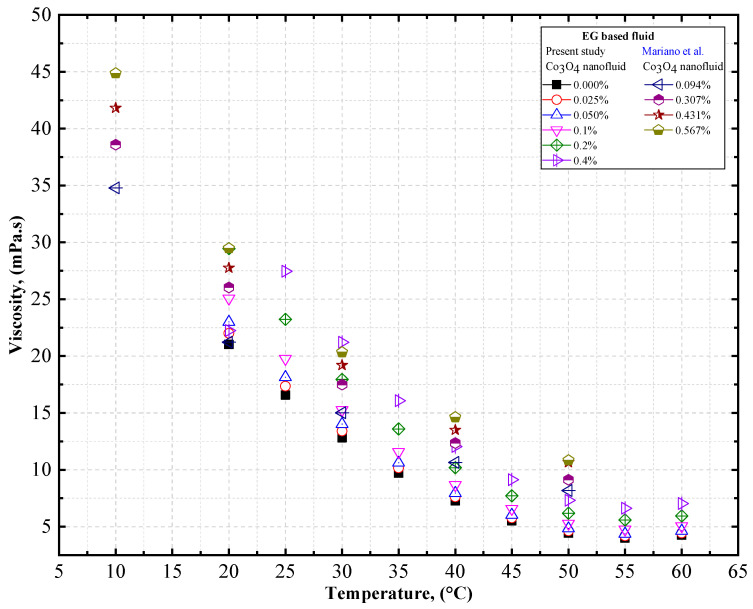
Comparison of experimental data on the viscosity of EG-based Co_3_O_4_ nanofluids. Reprinted with permission from Ref. [59], 2022, Elsevier.

To verify the accuracy of our measurements, the viscosity of EG-based Co_3_O_4_ nanofluids was measured at different temperatures, and was compared with the data obtained by Mariano et al. [59]. Figure 12 compares the experimental thermal conductivity of DW-based Co_3_O_4_ nanofluids derived from the present study with the data obtained by Mariano et al. [59] for Co_3_O_4_ nanofluids. Similar trends of increasing viscosity with an increase in the concentration of the solid volume fraction and decreasing viscosity with an increase in temperature was observed by Mariano et al. [59] for EG-based Co_3_O_4_ nanofluids (Table 12).

### 4.3. Comparison of the Theoretical Density, Thermal Conductivity, and Viscosity of Co_3_O_4_/DW, Co_3_O_4_/EG, Co_3_O_4_/60EG:40DW, Co_3_O_4_/40EG:60DW, and Co_3_O_4_/20EG:80DW Nanofluids

**Figure 13:**
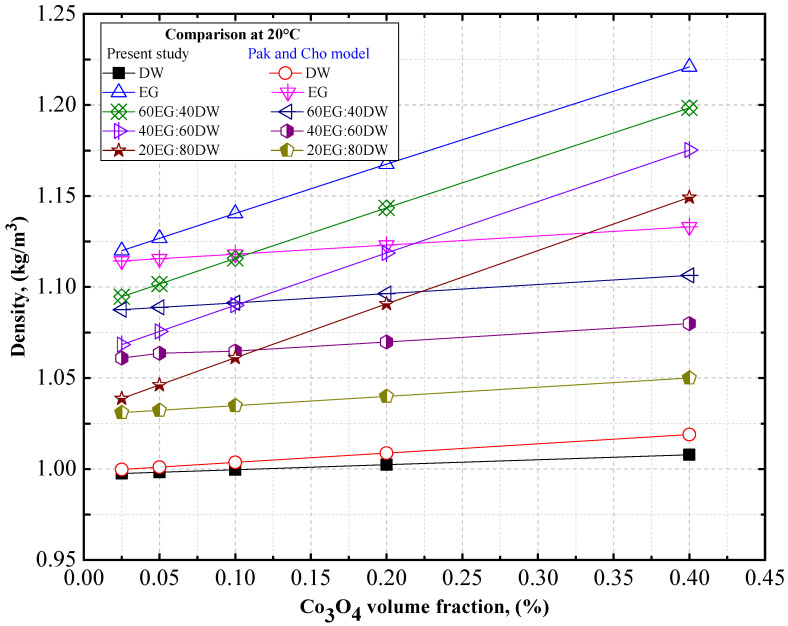
Density comparison between our experimental data and Pak and Cho’s [48] model for several solid volume fraction concentrations of Co_3_O_4_/DW, Co_3_O_4_/EG, Co_3_O_4_/60EG:40DW, Co_3_O_4_/40EG:60DW, and Co_3_O_4_/20EG:80DW nanofluids.

Figure 13 shows the density comparison of Co_3_O_4_/DW, Co_3_O_4_/EG, Co_3_O_4_/60EG:40DW, Co_3_O_4_/40EG:60DW, and Co_3_O_4_/20EG:80DW nanofluids with various solid volume fraction concentrations between the experimental data and Pak and Cho’s [48] model at 20 °C for Co_3_O_4_ nanofluids.

Similar trends of increasing density with an increase in the concentration of solid volume fraction and decreasing density with an increase in temperature were observed by Pak and Cho [48] for Co_3_O_4_/DW, Co_3_O_4_/EG, Co_3_O_4_/60EG:40DW, Co_3_O_4_/40EG:60DW, and Co_3_O_4_/20EG:80DW nanofluids.

**Figure 14:**
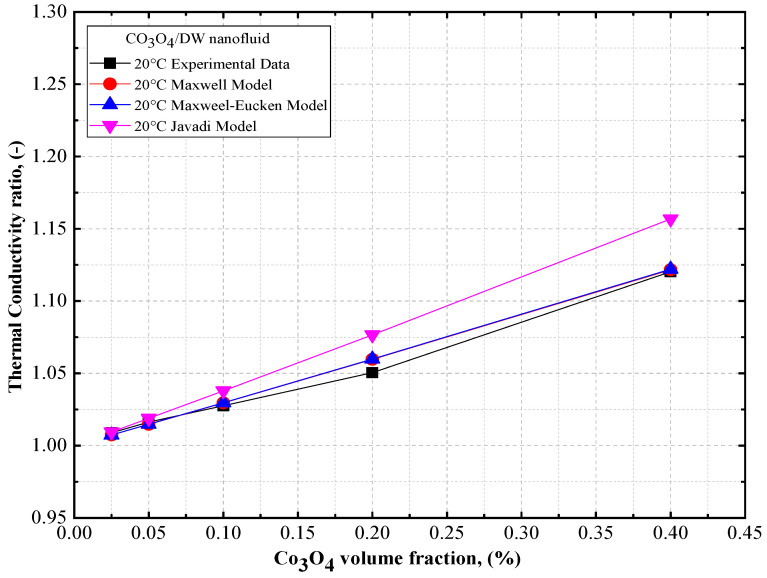
Comparison of the thermal conductivity ratios of DW-based Co_3_O_4_ nanofluids vs. theoretical models.

**Figure 15:**
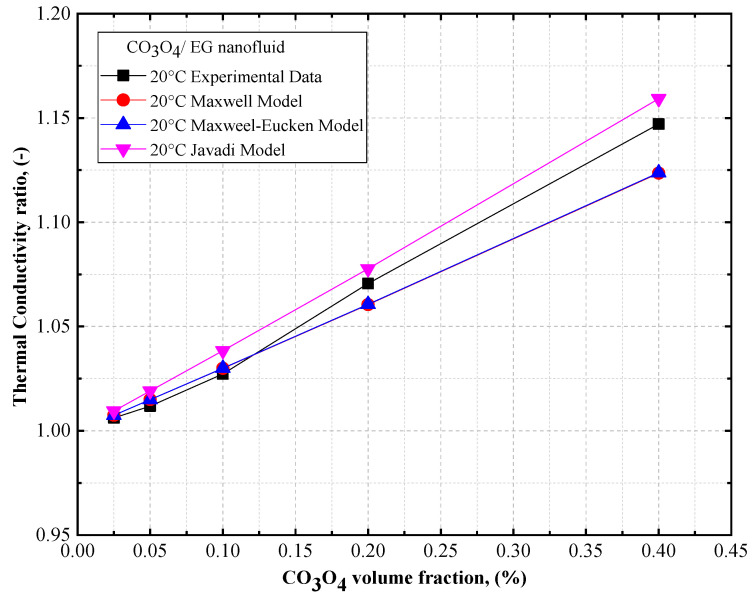
Comparison of the thermal conductivity ratios of EG-based Co_3_O_4_ nanofluids vs. theoretical models.

The thermal conductivity ratios of DW- and EG-based Co_3_O_4_ nanofluids are presented in Figure 14 and Figure 15, respectively, as a function of volume concentration, along with the theoretical models for the DW- and EG-based Co_3_O_4_ nanofluids. Based on Figure 14 and Figure 15, it can be observed that there was a similar trend in the thermal conductivity of the DW- and EG-based Co_3_O_4_ nanofluids (an increase in the concentration of the solid volume fraction) as observed using the Maxwell model [49], Maxwell–Eucken model [50], and Yu and Choi model [51] for Co_3_O_4_/DW and Co_3_O_4_/EG nanofluids. In this regard, a good agreement exists between the experimental results and the Maxwell model [49].

**Figure 16:**
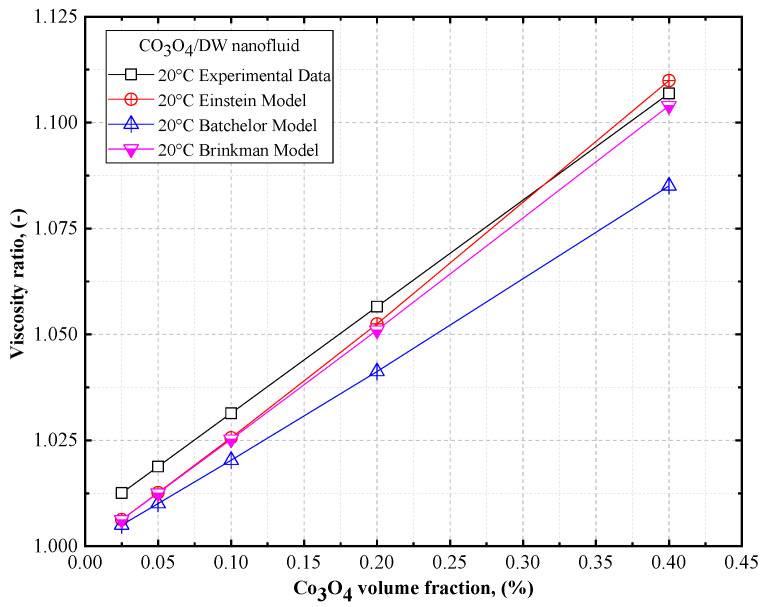
Comparison of the viscosity ratios of DW-based Co_3_O_4_ nanofluids vs. theoretical models.

**Figure 17:**
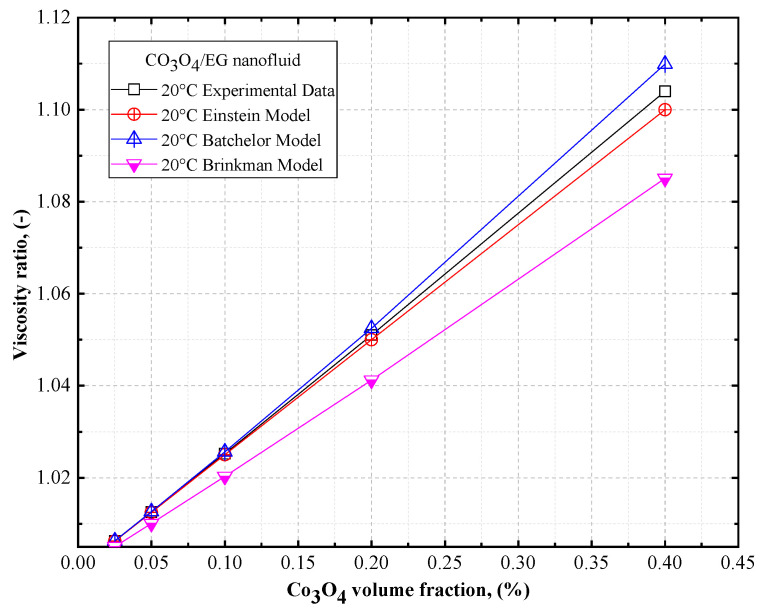
Comparison of the viscosity ratios of EG-based Co_3_O_4_ nanofluids vs. theoretical models.

The viscosity ratios of DW- and EG-based Co_3_O_4_ nanofluids are shown in Figure 16 and Figure 17, respectively, as a function of volume concentrations, along with the theoretical models for the DW- and EG-based Co_3_O_4_ nanofluids. Based on Figure 16 and Figure 17, it can be observed that there was a similar trend in the viscosity of the DW- and EG-based Co_3_O_4_ nanofluids with an increase in the concentration of the solid volume fraction as observed using the Einstein model [52], Brinkman model [53], and Batchelor model [54] for Co_3_O_4_/DW and Co_3_O_4_/EG nanofluids. Thus, a good agreement exists between the experimental results and the Einstein model [52].

## 5. Conclusions

In the present study, the thermophysical properties of the Co_3_O_4_ nanoparticles suspended in distilled water were experimentally tested in a laboratory at Al-Hussein Bin Talal University. The nanofluids were prepared at volume concentrations of 0.025, 0.05, 0.1, 0.2, and 0.4 vol.%, within a temperature range from 20 °C to 60 °C.

1. The density of the Co_3_O_4_/DW, Co_3_O_4_/EG, and Co_3_O_4_/DW–EG mixture nanofluids decreased with increasing temperature, whereas it increased with increasing particle volume concentration. The lowest and highest density values were confirmed for the 0.025 vol.% Co_3_O_4_/DW nanofluid at 20 °C and the 0.4 vol.% Co_3_O_4_/EG60:DW40 at 60 °C, respectively.
2. The density enhancement was about 0.73% and 11.48% for the temperatures 20 °C and 60 °C, respectively, compared to the base fluid (EG20:DW80).
3. The viscosity of the Co_3_O_4_/DW, Co_3_O_4_/EG, and Co_3_O_4_/EG–DW mixture nanofluids also showed a similar variation trend to that of the density.
4. The viscosity enhancement was about 0.70% and 1.61% at temperatures of 20 °C and 60 °C, respectively, compared to the base fluid (EG40:DW60).
5. The thermal conductivity of the Co_3_O_4_/DW, Co_3_O_4_/EG, and Co_3_O_4_/DW–EG mixture nanofluids increased with temperature and particle volume concentration. The lowest and highest density values were confirmed for the 0.025 vol.% Co_3_O_4_/DW nanofluid and the 0.4 vol.% Co_3_O_4_/EG60:DW40 nanofluid, respectively.
6. The thermal conductivity of the Co_3_O_4_/EG60:DW40 nanofluid (at a 0.4% volume concentrations) was enhanced by about 0.16% and 1.17% at temperatures of 20 °C and 60 °C, respectively, compared to the base fluid (EG60:DW40).
7. The maximum thermal conductivity of 10.8% prevailed at 20 °C at the volume fraction of 0.4%, and the maximum viscosity of 7.45% prevailed at 20 °C at the volume fraction of 0.4%.

## Figures and Tables

**Table 1:** Characteristics of cobalt oxide (Co_3_O_4_) nanoparticles and base fluids at 20 °C. Reprinted with permission from Ref. [42], 2022, Elsevier.

Characteristics	Co_3_O_4_	EG	DW	60EG:40DW	40EG:60DW	20EG:80DW
Purity (%)	99.5	99	(-)	(-)	(-)	(-)
Black	color	(-)	(-)	(-)	(-)	(-)
Particle size measurement (nm)	≤50	(-)	(-)	(-)	(-)	(-)
Density (g/cm^3^)	6.11	1.113	0.9985	1.08627	1.05968	1.02972
Viscosity (mPa.s)	(-)	21	0.89	5.38	2.96	1.65
Thermal conductivity (W/mK)	69	0.258	0.602	0.334	0.404	0.492

**Table 2:** Coefficients *Ai* of Equations (2) and (3).

*ρ*	*A*_1_	*A*_2_	*A*_3_	*A*_4_	*A*_5_	*A*_6_
1	1127.68	−0.65816	−6.1765 × 10^−4^	0.30590	0.13781	−1.8961 × 10^−3^
2	1132.35	−0.67950	−4.7565 × 10^−4^	0.90820	−0.26348	−3.3787 × 10^−3^
3	1139.48	0.71040	−4.3663 × 10^−4^	1.1712	−0.52694	−3.8797 × 10^−3^

**Table 3:** The mass of cobalt oxide required in different particle volume concentrations.

∅, (%)	Mass of Co_3_O_4_ Required (*g*) for Several Base Fluids				
	EG	DW	60EG:40W	40EG:60W	20EG:80W
0.025	0.068604	0.076471	0.071548	0.073117	0.074756
0.05	0.136555	0.152215	0.142416	0.145539	0.148802
0.10	0.274209	0.305653	0.274209	0.274209	0.274209
0.20	0.547869	0.610694	0.571381	0.583911	0.597002
0.40	1.093542	1.218940	1.140472	1.165481	1.191612

**Table 4:** Maximum uncertainty in experimental parameters and measurement devices.

Parameters	Type	Uncertainty (%)
Density (*ρ*)	Pycnometer	±0.2
Velocity (*μ*)	A&D Vibro Viscometer (SV-10)	±1
Thermal conductivity (*k*)	KD2-Pro	±5
Temperature (*T*) thermostat	Memmert SV 14–22	±0.1

**Table 5:** Conductivity meter properties.

Characteristics	Value
KD2 sensor:	Model: KS-1
Limit of measurement:	0.02 to 2 (W/mK)
Accuracy:	5% from 0.2–2 (W/mK)

**Table 6:** Experimental density of Co_3_O_4_/EG, DW, and EG–DW mixture nanofluids.

∅	Test Temperature (°C)								
	20 °C	25 °C	30 °C	35 °C	40 °C	45 °C	50 °C	55 °C	60 °C
**Co_3_O_4_/DW**									
0.025	0.9985	0.9974	0.9964	0.9953	0.9943	0.9932	0.9922	0.9911	0.9901
0.05	0.9991	0.9981	0.9971	0.9960	0.9950	0.9939	0.9929	0.9918	0.9908
0.1	1.0005	0.9995	0.9984	0.9974	0.9963	0.9953	0.9942	0.9932	0.9921
0.2	1.0033	1.0022	1.0012	1.0001	0.9991	0.9980	0.9970	0.9959	0.9949
0.4	1.0087	1.0077	1.0066	1.0056	1.0045	1.0035	1.0024	1.0014	1.0004
**Co_3_O_4_/EG**									
0.025	1.1199	1.1164	1.1128	1.1092	1.1056	1.1019	1.0982	1.0945	1.0907
0.05	1.1268	1.1233	1.1197	1.1161	1.1125	1.1089	1.1052	1.1015	1.0978
0.1	1.1404	1.1369	1.1334	1.1299	1.1264	1.1228	1.1192	1.1155	1.1119
0.2	1.1675	1.1641	1.1607	1.1573	1.1539	1.1504	1.1469	1.1434	1.1398
0.4	1.2209	1.2177	1.2145	1.2113	1.2081	1.2049	1.2016	1.1983	1.1949
**Co_3_O_4_/20EG:80DW**									
0.025	1.0387	1.0369	1.0350	1.0330	1.0309	1.0291	1.0262	1.0237	1.0211
0.05	1.0462	1.0444	1.0426	1.0406	1.0385	1.0367	1.0339	1.0314	1.0288
0.1	1.0611	1.0594	1.0576	1.0556	1.0535	1.0518	1.049	1.0466	1.0440
0.2	1.0908	1.0891	1.0873	1.0855	1.0834	1.0818	1.0791	1.0767	1.0743
0.4	1.1492	1.1477	1.1460	1.1443	1.1424	1.1409	1.1384	1.1362	1.1339
**Co_3_O_4_/40EG:60DW**									
0.025	1.0683	1.0662	1.0640	1.0616	1.0590	1.0563	1.0535	1.0505	1.0473
0.05	1.0756	1.0735	1.0712	1.0689	1.0663	1.0637	1.0608	1.0579	1.0547
0.1	1.0900	1.0879	1.0857	1.0834	1.0809	1.0783	1.0755	1.0726	1.0695
0.2	1.1187	1.1167	1.1146	1.1123	1.1099	1.1073	1.1047	1.1018	1.0989
0.4	1.1752	1.1733	1.1714	1.1692	1.1670	1.1646	1.1621	1.1595	1.1568
**Co_3_O_4_/60EG:40DW**									
0.025	1.0946	1.0922	1.0897	1.0871	1.0844	1.0815	1.0785	1.0754	1.0722
0.05	1.1017	1.0993	1.0968	1.0942	1.0915	1.0886	1.0857	1.0826	1.0794
0.1	1.1157	1.1133	1.1109	1.1083	1.1057	1.1029	1.1000	1.0969	1.0938
0.2	1.1435	1.1412	1.1389	1.1364	1.1338	1.1311	1.1283	1.1254	1.1223
0.4	1.1984	1.1963	1.1941	1.1918	1.1893	1.1868	1.1842	1.1815	1.1786

**Table 7:** Experimental viscosity of Co_3_O_4_/EG, DW, and EG–DW mixture nanofluids.

∅	Test Temperature (°C)								
	20 °C	25 °C	30 °C	35 °C	40 °C	45 °C	50 °C	55 °C	60 °C
**Co_3_O_4_/DW**									
0.025	1.04	0.92	0.82	0.73	0.66	0.59	0.54	0.5	0.47
0.05	1.06	0.94	0.83	0.74	0.67	0.61	0.55	0.51	0.48
0.1	1.10	0.97	0.86	0.77	0.69	0.63	0.57	0.53	0.49
0.2	1.18	1.04	0.93	0.83	0.74	0.67	0.62	0.57	0.53
0.4	1.34	1.18	1.05	0.94	0.84	0.76	0.7	0.64	0.60
**Co_3_O_4_/EG**									
0.025	22.00	17.35	13.40	10.20	7.61	5.77	4.62	4.18	4.44
0.05	23.00	18.14	14.01	10.60	7.96	6.03	4.83	4.37	4.64
0.1	25.07	19.77	15.27	11.60	8.67	6.57	5.27	4.76	5.06
0.2	29.45	23.23	17.94	13.60	10.2	7.72	6.19	5.60	5.94
0.4	34.82	27.46	21.21	16.10	12.00	9.12	7.32	6.62	7.03
**Co_3_O_4_/20EG:80DW**									
0.025	9.42	7.49	5.85	4.50	3.44	2.66	2.17	1.97	2.06
0.05	9.84	7.82	6.10	4.69	3.58	2.77	2.27	2.06	2.14
0.1	10.69	8.49	6.62	5.09	3.88	3.01	2.45	2.22	2.32
0.2	12.49	9.92	7.73	5.93	4.52	3.49	2.84	2.58	2.70
0.4	14.73	11.69	9.11	6.99	5.32	4.11	3.34	3.03	3.17
**Co_3_O_4_/40EG:60DW**									
0.025	13.62	10.78	8.368	6.38	4.83	3.70	2.99	2.71	2.85
0.05	14.23	11.26	8.741	6.67	5.04	3.86	3.12	2.83	2.98
0.1	15.48	12.25	9.509	7.25	5.48	4.19	3.39	3.07	3.23
0.2	18.14	14.35	11.13	8.49	6.41	4.90	3.96	3.59	3.78
0.4	21.42	16.95	13.15	10	7.56	5.78	4.67	4.23	4.46
**Co_3_O_4_/60EG:40DW**									
0.025	17.81	14.06	10.89	8.27	6.22	4.73	3.81	3.44	3.65
0.05	18.61	14.7	11.38	8.64	6.5	4.94	3.98	3.60	3.81
0.1	20.28	16.01	12.39	9.41	7.08	5.38	4.33	3.92	4.15
0.2	23.8	18.79	14.54	11.00	8.30	6.31	5.07	4.59	4.86
0.4	28.12	22.2	17.18	13.00	9.80	7.45	5.99	5.42	5.74

**Table 8:** Experimental thermal conductivity for Co_3_O_4_/EG, DW, and EG–DW mixture nanofluids.

∅	Test Temperature (°C)								
	20 °C	25 °C	30 °C	35 °C	40 °C	45 °C	50 °C	55 °C	60 °C
**Co_3_O_4_/DW**									
0.025	0.611	0.620	0.629	0.638	0.647	0.655	0.664	0.672	0.680
0.05	0.621	0.630	0.639	0.648	0.657	0.666	0.674	0.683	0.691
0.1	0.641	0.650	0.660	0.669	0.678	0.687	0.696	0.705	0.713
0.2	0.678	0.688	0.698	0.708	0.717	0.727	0.736	0.745	0.754
0.4	0.750	0.761	0.772	0.783	0.794	0.804	0.814	0.824	0.834
**Co_3_O_4_/EG**									
0.025	0.260	0.261	0.261	0.262	0.262	0.263	0.263	0.264	0.264
0.05	0.261	0.262	0.263	0.263	0.264	0.264	0.265	0.265	0.266
0.1	0.265	0.266	0.267	0.267	0.268	0.268	0.269	0.269	0.27
0.2	0.277	0.277	0.278	0.279	0.279	0.28	0.28	0.281	0.281
0.4	0.296	0.297	0.298	0.298	0.299	0.300	0.300	0.301	0.301
**Co_3_O_4_/20EG:80DW**									
0.025	0.471	0.476	0.482	0.488	0.493	0.498	0.504	0.509	0.514
0.05	0.477	0.483	0.489	0.494	0.500	0.505	0.511	0.516	0.521
0.1	0.491	0.497	0.503	0.508	0.514	0.520	0.525	0.531	0.536
0.2	0.517	0.524	0.530	0.536	0.542	0.548	0.554	0.559	0.565
0.4	0.568	0.575	0.582	0.589	0.596	0.602	0.609	0.615	0.621
**Co_3_O_4_/40EG:60DW**									
0.025	0.400	0.404	0.408	0.412	0.416	0.42	0.424	0.427	0.431
0.05	0.405	0.409	0.413	0.417	0.421	0.425	0.429	0.432	0.436
0.1	0.416	0.420	0.424	0.428	0.432	0.436	0.440	0.443	0.447
0.2	0.437	0.441	0.446	0.450	0.454	0.459	0.463	0.467	0.470
0.4	0.478	0.483	0.487	0.492	0.497	0.501	0.506	0.510	0.514
**Co_3_O_4_/60EG:40DW**									
0.025	0.472	0.476	0.482	0.488	0.493	0.498	0.504	0.509	0.514
0.05	0.478	0.483	0.489	0.494	0.500	0.505	0.511	0.516	0.521
0.10	0.492	0.497	0.503	0.508	0.514	0.52	0.525	0.531	0.536
0.20	0.519	0.524	0.530	0.536	0.542	0.548	0.554	0.559	0.565
0.40	0.570	0.575	0.582	0.589	0.596	0.602	0.609	0.615	0.621

**Table 9:** Variations in the thermal conductivity ratio as a function of solid volume fraction and temperature.

∅	Thermal Conductivity Ratio			
	30 °C	40 °C	50 °C	60 °C
0.1 vol%	1.056	1.059	1.160	1.170
0.2 vol%	1.100	1.107	1.195	1.200
0.3 vol%	1.082	1.133	1.199	1.230
0.4 vol%	1.160	1.253	1.236	1.255

**Table 10:** Variations in relative viscosity (*μ_rel_*) as a function of solid volume fraction and temperature.

∅	Relative Viscosity			
	30 °C	40 °C	50 °C	60 °C
0.1 vol%	1.140	1.045	1.160	1.170
0.2 vol%	1.100	1.020	1.130	1.340
0.3 vol%	1.160	1.17	1.150	1.170
0.4 vol%	1.310	1.190	1.160	1.180

**Table 11:** Variation in thermal conductivity as a function of solid volume fraction and temperature.

∅	Thermal Conductivity (Wm^−1^k^−1^)		
	283.15 K	303.15 K	323.15 K
0.0094	0.263	0.261	0.259
0.0307	0.286	0.284	0.283
0.0431	0.297	0.296	0.294
0.0567	0.309	0.307	0.306

**Table 12:** Variation in viscosity as a function of solid volume fraction and temperature.

∅	Viscosity (mPa.s)				
	283.15 K	293.15 K	303.15 K	313.15 K	323.15 K
0.0094	34.79	22.24	15.01	10.64	8.17
0.0307	38.59	26.04	17.49	12.35	9.12
0.0431	41.82	27.75	19.20	13.49	10.64
0.0570	44.86	29.48	20.34	14.63	10.83

## Data Availability

Not applicable.
